# A comparison between a hinged plate and screw system and a conventional tension-band plate and screw system used for correction of an angular deformity of the lower limb: an animal study

**DOI:** 10.1186/s13018-015-0198-4

**Published:** 2015-05-03

**Authors:** Zhenkai Wu, Dahang Zhao, Li Zhao, Jianlin Liu, Hai Li, Jie Zhu, Fengcang Ma, Daniel Edward Porter

**Affiliations:** Department of Pediatric Orthopaedics, Xin-Hua Hospital affiliated to Shanghai Jiao Tong University School of Medicine, No. 1665, Kongjiang Road, Shanghai, 200092 China; Department of Orthopaedic Surgery, Edinburgh Orthopaedic Trauma Unit Royal Infirmary of Edinburgh, Edinburgh, EH16 4SU UK; School of Materials Science & Engineering, University of Shanghai for Science and Technology, No. 516, Jungong Road, Shanghai, Shanghai 200093 China

**Keywords:** Temporary hemiepiphysiodesis, Tension-band plate and screw system, Staple, Hinged plate and screw system, Angular deformity

## Abstract

**Background:**

The purpose of the animal study is to introduce a newly designed hinged plate and screw system for correction of angular deformities of the lower limbs. The technique was compared with the use of a conventional tension-band plate and screw system.

**Methods:**

This is a randomized controlled animal trial. Eight 3-month-old Bama miniature pigs were used to establish animal models. In each animal, one leg was randomly allocated into study group and another leg into control group. Legs of the study group were corrected with a hinged plate and screw system, and legs of the control group were corrected with a conventional tension-band plate and screw system. The corrective rates of medial slope angle, medial proximal tibial angle, and angle of the two arms of the hinged plate were measured. Residual stress on the implants was also evaluated. A *P* < 0.05 was statistical significant.

**Results:**

At the final measurements of 18 weeks, the mean corrective rates of medial slope angle, medial proximal tibial angle, and angle of the two arms of the study group were 0.71°/week, 0.85°/week, and 2.18°/week, respectively; the data in the control group were 0.84°/week, 0.89°/week, and 2.13°/week, respectively. No significant difference was found between the groups regarding the mean corrective rates of the angles (*P* < 0.05). The mean residual stress in the study group was 643.35 MPa, and measurement in the control group was 1,273.63 MPa, with a significant difference (*P* < 0.05).

**Conclusions:**

Compared to the conventional tension-band plate and screw system, the hinged plate and screw system may be more reliable for correction of angular deformities of the lower limb.

## Introduction

Angular deformities of the lower limb may be congenital or acquired in children, and the treatments are still challenging problems [1]. Temporary hemiepiphysiodesis with an implant to arrest the growth of the bone is an effective method to correct the deformities, but appropriate implant selection is still controversial [1-3].

In 1949, Blount et al. [2] developed the epiphyseal stapling technique to correct angular deformities by controlling bone growth. The technique is effective, but systematical failure often occurs due to weak support of the staples. In 2006, Stevens [3] developed a tension-band plate and screw system to reduce fixation failure. The system is stronger and now commonly used in clinical practices, resulting in success rates of 78.5% to 94.1% [4-10]. However, that straight plate rarely matches the convex contour of the physis and metaphysic. The poorly mounted implant system is relatively unstable and may produce nonuniform stress distribution on the growth plate and stress concentration on the screws [11]. Thus, fixation failure may happen, especially in patients with abnormal physis [12]. In a series of 31 cases, Schroerlucke reported 26% of fixation failure in the treatment of angular deformities of the lower limb [13]. In addition, the middle portion of the straight plate may produce excessive compression on the periosteum and perichondrium over the convex surface of the physis and epiphysis, resulting in unpredictable complications [14].

**Figure 1:**
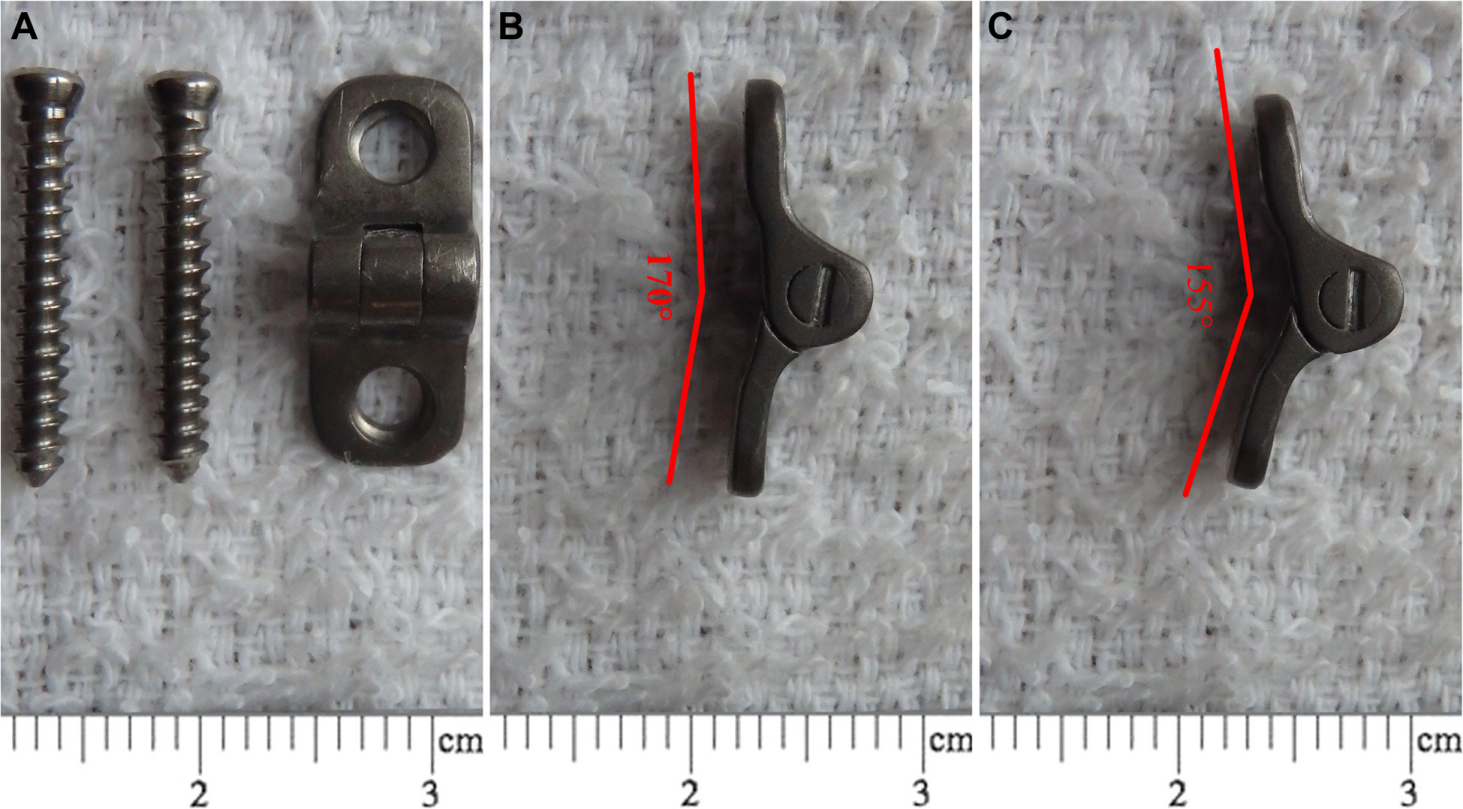
The newly designed hinged plate and screw system. **(A)** The plate has a hinge and two arms and two 2 mm diameter screws. **(B)** Maximal angle of the two arms. **(C)** Minimal angle of the two arms.

**Figure 2:**
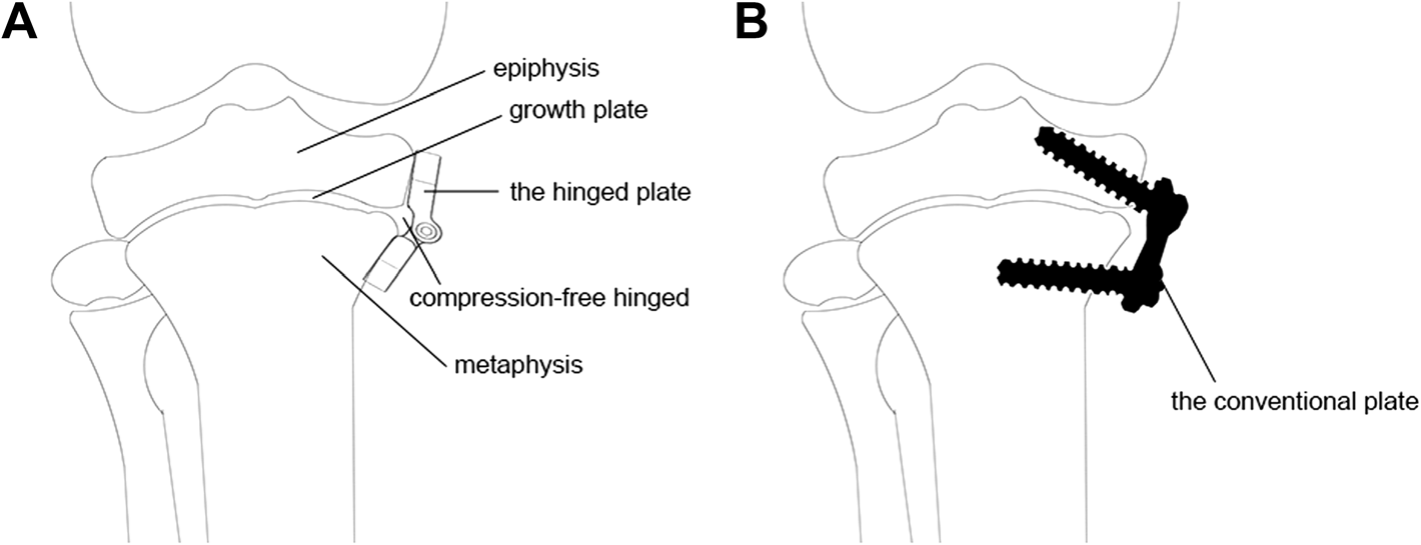
Sketch map of hemiepiphysiodesis. **(A)** The hinged plate almost completely matches the contour of the bone. **(B)** The straight conventional plate does not match the bone surface.

The purpose of the study was to introduce a hinged plate and screw system for correction of angular deformities of the lower limb in animal models (Figures 1 and 2). We also compared the use of the implant with the use of the conventional tension-band plate and screw system in the experimental cohort study.

## Materials and methods

From August 2013 to January 2014, nine 3-month-old Bama miniature pigs were used to establish animal models. All the animals were male. In each animal, one researcher randomly allocated one leg into study group and another leg into control group. The random method was flipping a coin. This study was approved by the Institutional Review Board/Ethics Committee of Xin-Hua Hospital.

### Anesthesia and operative techniques

The animals were fasted for 24 hours prior to surgery and were sedated with an intramuscular injection of diazepam and 3% pentobarbital sodium (2 ml/kg). Operations were performed under general anesthesia by intravenous propofol (10 mg/kg/h) and fentanyl (25 mg/kg/h). Cefazolin Sodium (Sinopharm Chemical Reagent Co., Ltd, Shanghai, China) was injected prior to skin incision and every morning for the following 3 days. Postoperative pain was managed with tramadol.

#### Study group

**Figure 3:**
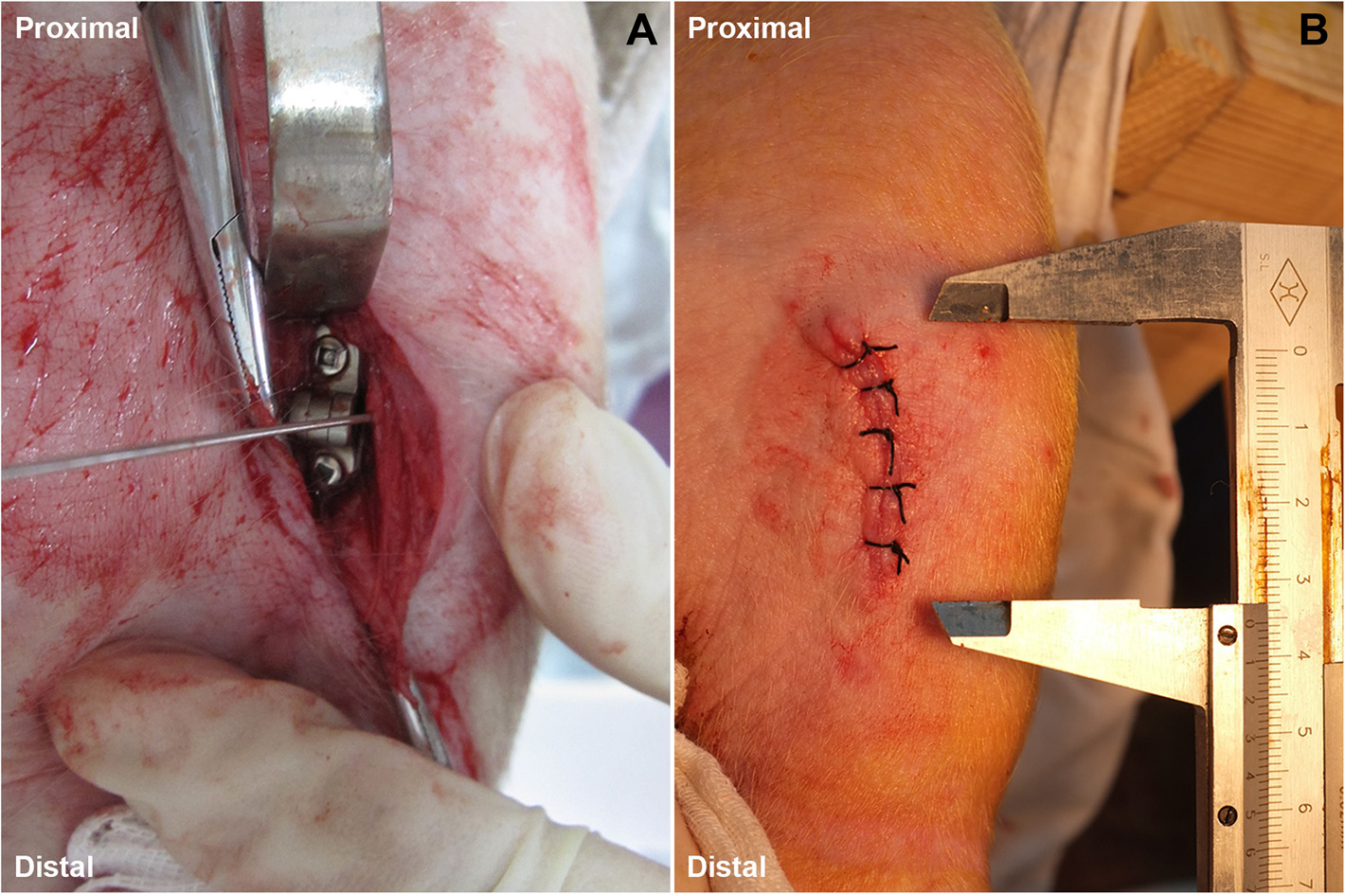
Intraoperative photos. **(A)** A hinged plate and screw system is implanted. **(B)** No obvious prominence observed after wound closure.

The animal was placed in the supine position on a radiolucent table. A 2–3-cm-long incision was made on the medial aspect of the proximal tibia (Figure 3). Dissection went deep to the tibia with preservation of the periosteum. In the mid-coronal plane, a 1-mm Keith needle was drilled into the physis. That procedure was performed under guidance of fluoroscope. A hinged plate (HP group, Shanghai Puwei Medical instrument Co., Ltd., Shanghai, China) was placed extra-periosteally and mounted over the needle to center it upon the physis. The cortex was predrilled with a 1.8-mm drill bit so as to reduce stress during screw insertion [3,4]. The screws should be placed parallel or slightly convergent (0° to 20°) without encroaching on the growth plate cartilage. The two screws were tightened alternately.

#### Control group

As described by Stevens [3], surgical procedures were performed using a tension-band plate and screw system (CP group, Shanghai Puwei Medical instrument Co., Ltd., Shanghai, China).

### Postoperative managements

**Figure 4:**
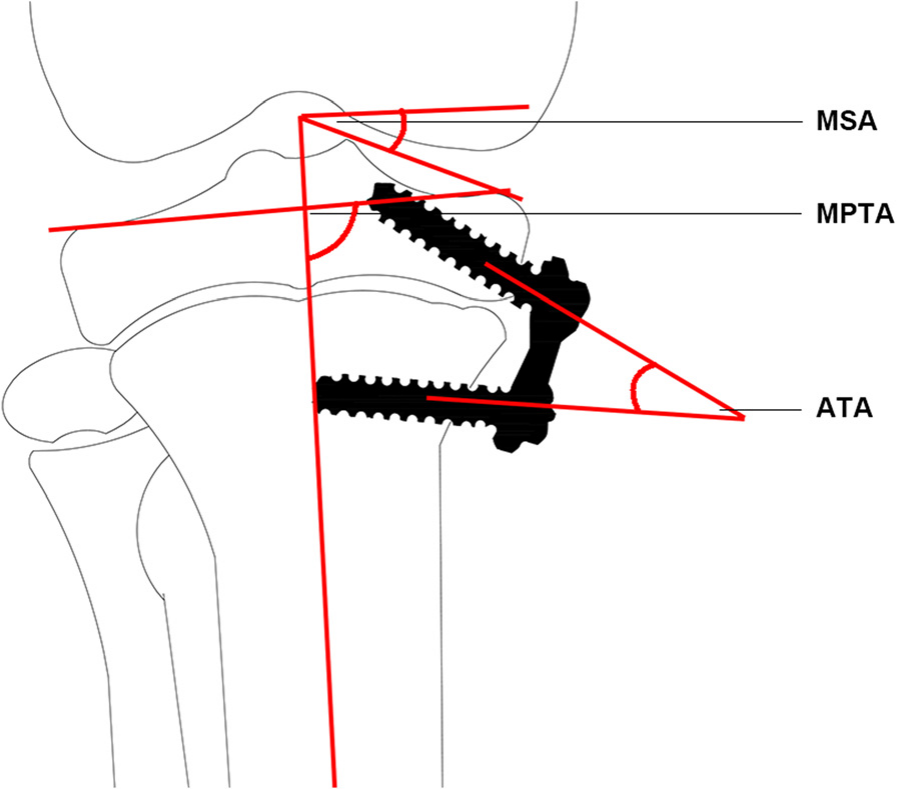
Measurements on standard anterioposterior radiograph of the knee joint. MSA, medial slope angle; MPTA, medial proximal tibial angle; and ATA, angle of the two arms.

Immediately after operation, we assessed the medial slope angle (MSA, the angle formed between the line vertical to the anatomical axis of the tibia and the line tangent to the medial tibial plateau), the medial proximal tibial angle (MPTA, the angle formed between the anatomical axis of the tibia and the line drawn between the medial and lateral corners of the tibial plateau), and the angle of the two arms (ATA) on anterioposterior fluoroscopic images. Radiographs were also obtained at the end of 6, 12, and 18 weeks postoperatively (Figure 4). Immediately after the last radiography, the animal was scarified and the implant was removed. The epiphysis and metaphysis with the periosteal and perichondral beneath the implant, as well as the adjacent epiphyseal growth plate and metaphysic, were harvested. The sample was fixed in 10% formalin and embedded in methyl methacrylate. In the frontal plane, the sample was cut into 7-mm-thick sections, and the sections were stained with safranin-O/fast green (Sinopharm Chemical Reagent Co., Ltd, Shanghai, China).

### Measurements of the samples

All measurements were conducted in a blinded fashion. The measurements were performed by two of the authors independently, and the mean value of the two measurements was used for analysis. The agreement between the two authors was evaluated with the Spearman correlation coefficient for interrater agreement and intraclass correlation coefficient.

**Figure 5:**
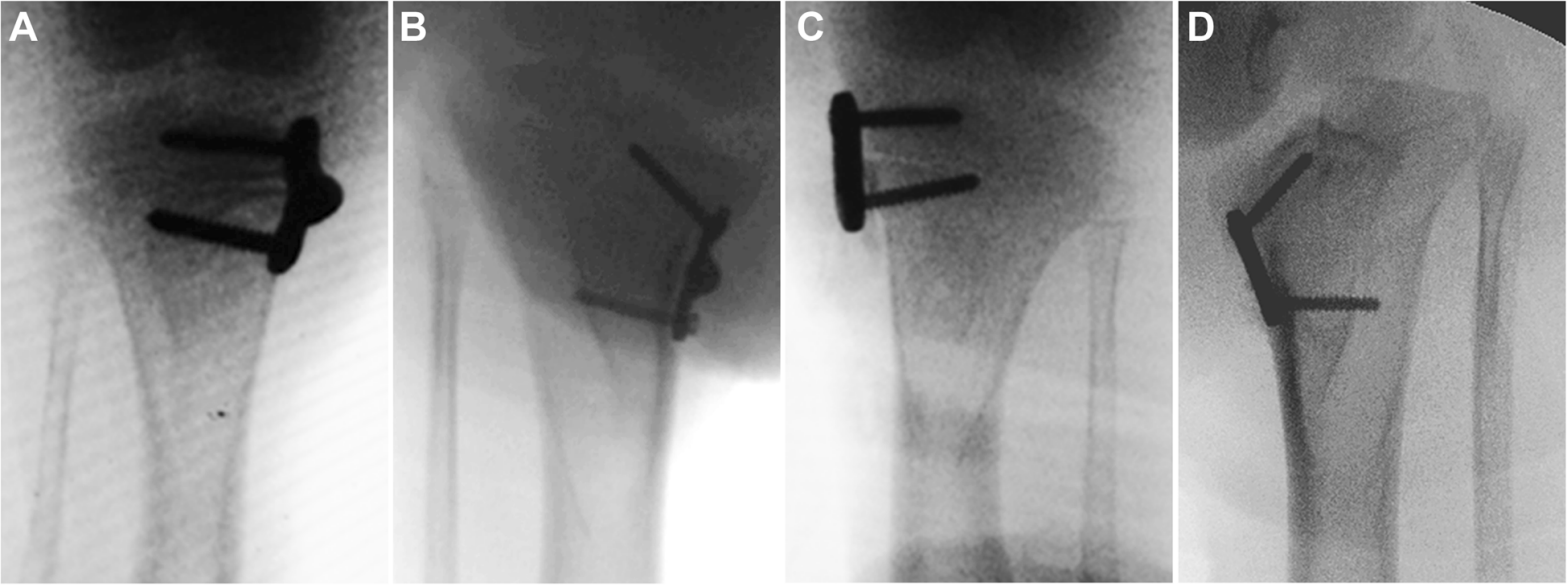
Standard anterioposterior radiographs of the tibia. **(A)** Immediately after correction with a hinged plate and screw system. **(B)** Eighteen weeks after operation. **(C)** Immediately after correction with a conventional plate and screw system. **(D)** Eighteen weeks after operation.

Efficacy of hemiepiphysiodesis was assessed with the method described by Kanellopoulos et al. [15]. On anterioposterior radiograph, we measured three angles, i.e., the MSA, MPTA, and ATA (Figure 5).

Residual stress (remaining stress of the sample after implant removal) at the rim of the metaphyseal screw hole was measured using X-ray diffraction (XRD, Bruker D8 Advance, Karlsruhe, Germany) [16,17]. Reflection of titanium alloy was set at 2theta = 70.631. Lattice strains were measured at six positions (psi values: 0.00, 11.5, 16.4, 20.3, 23.6, and 26.6). Residual stress was calculated using Leptos (Version 7.0, Bruker-AXS, Karlsruhe, Germany) according to the strains at six positions [16,17]. For histological study, the width of perichondrial tissue between the center of the plate and physis was measured on the 40 × magnified image of each section.

### Analysis

Statistical analysis was made using the paired *t*-test for comparing the values of all measurement parameters between the groups at the same interval, and 95% confidence intervals for pairwise differences were calculated. The level of statistical significance was set at *P* < 0.05.

## Results

One sample of the control group was excluded because of fixation failure at 6 weeks postoperatively. That animal was excluded. One animal had a superficial infection and recovered after 1-week application of antibiotics.

**Figure 6:**
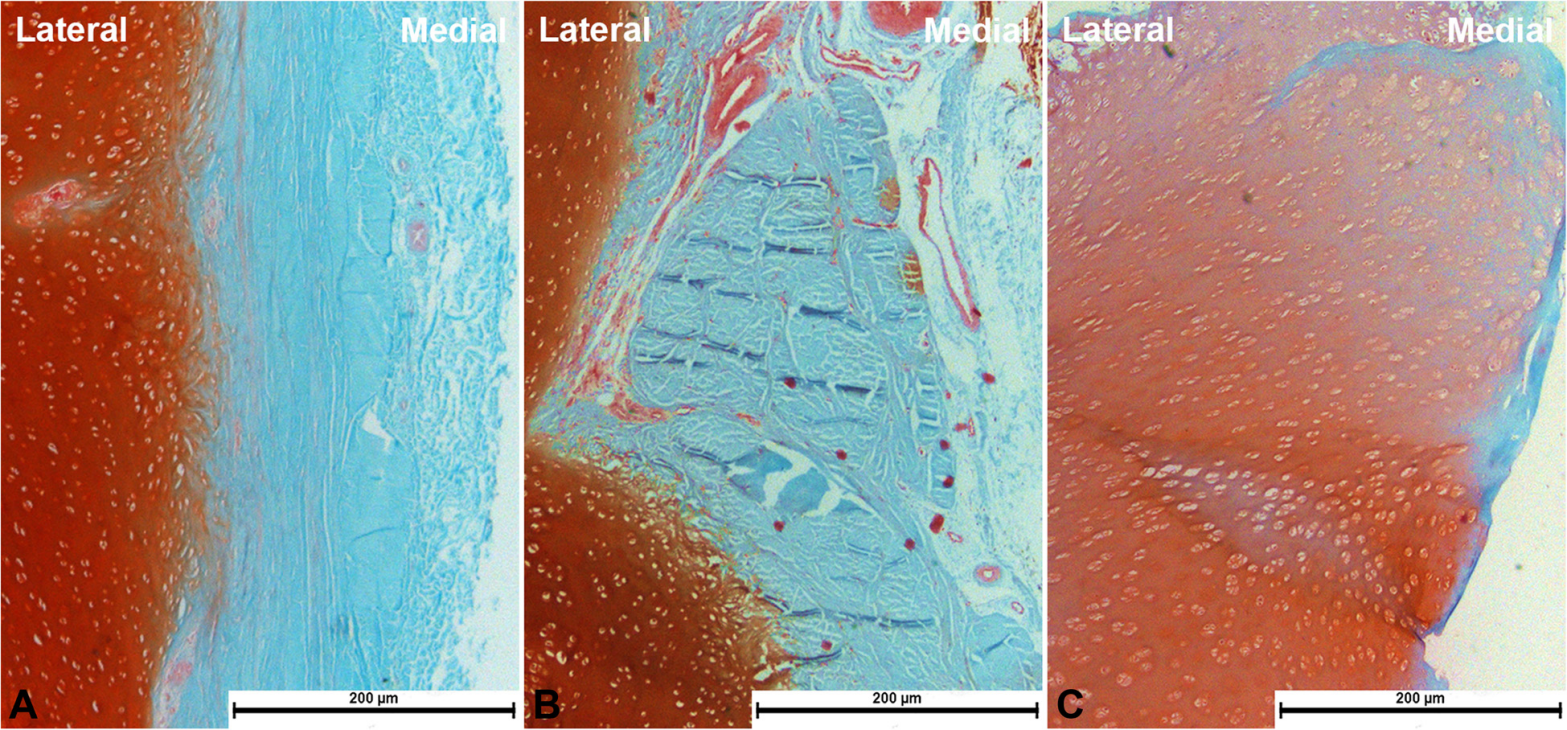
Images of histological sections. The bluish green area shows periosteal and perichondral (Safranin-O/fast green stain; ×40). **(A)** A section obtained from the tibia of a 3-month-old Bama miniature pig. The pig used for comparison did not have an operation. **(B)** The periosteal and perichondral beneath the hinged plate are intact after 18 weeks. **(C)** The periosteal and perichondral beneath the conventional tension-band plate are almost vanished after 18 weeks.

In the study group, the angles of MSA, MPTA, and ATA at 6, 12, and 18 weeks were shown in Table 1. The corrective rates of the angles of MSA, MPTA, and ATA were 0.71°/week, 0.85°/week, and 2.18°/week, respectively. The residual stress at the rim of metaphyseal screw hole was 643.35 ± 47.24 MPa (Table 1). The periosteum and perichondrium (stained bluish green) were deformed but were still intact (Figure 6). The mean width of perichondrial tissue was 155.38 ± 8.34 μm.

**Table 1:** **Demographics and measurement values of study group**

		**0 week**			**6 weeks**			**12 weeks**			**18 weeks**			
**Case**	**Side**	**MSA(°)**	**MPTA(°)**	**ATA (°)**	**MSA(°)**	**MPTA(°)**	**ATA(°)**	**MSA(°)**	**MPTA(°)**	**ATA(°)**	**MSA(°)**	**MPTA(°)**	**ATA(°)**	**RS(MPa)**
1	L	20	88	−20	25	85	−14	29	81	12	32	80	26	813.9
2	R	26	89	−12	27	87	5	29	77	22	31	67	32	681.9
3	L	22	93	−4	25	91	12	36	80	24	40	71	30	718.7
4	L	24	90	−4	25	90	6	27	85	15	33	81	26	465.9
5	R	22	90	−13	28	90	−2	34	85	20	40	76	34	415.9
6	R	22	98	0	22	95	7	24	86	28	33	75	31	703.9
7	R	21	94	−12	23	90	8	30	85	18	33	78	31	678.5
8	L	22	85	−5	24	81	11	29	81	19	31	76	33	668.1
Mean		22	91	−9	25	89	4	30	83	20	34	76	30	643.35

ATA, angle of the two arms; MPTA, medial proximal tibial angle; MSA, medial slope angle; RS, residual stress.

In the control group, the angles of MSA, MPTA, and ATA at 6, 12, and 18 weeks were shown in Table 2. The corrective rates of the angles of MSA, MPTA, and ATA were 0.84°/week, 0.89°/week, and 2.13°/week, respectively. The residual stress at the rim of metaphyseal screw hole was 1,273.63 ± 132.28 MPa (Table 2). The periosteum and perichondrium (stained bluish green) were disorganized and damaged (Figure 6). The mean width of perichondrial tissue was 61.83 ± 7.56 μm.

**Table 2:** **Demographics and measurement values of control group**

		**0 week**			**6 weeks**			**12 weeks**			**18 weeks**			
**Case**	**Side**	**MSA(°)**	**MPTA(°)**	**ATA (°)**	**MSA(°)**	**MPTA(°)**	**ATA(°)**	**MSA(°)**	**MPTA(°)**	**ATA(°)**	**MSA(°)**	**MPTA(°)**	**ATA(°)**	**RS(MPa)**
1	R	21	91	−1	26	84	17	32	77	29	35	72	32	1,441.2
2	L	13	85	0	22	83	4	23	80	22	28	75	33	1,318.7
3	R	23	89	−17	29	89	6	37	84	27	37	79	33	1,450.9
4	R	20	89	−1	25	88	4	30	86	14	40	83	30	1,765.5
5	L	20	89	−8	31	82	19	37	79	26	41	71	32	651.4
6	L	22	97	−1	31	91	15	34	80	31	30	65	33	1,235.1
7	L	20	94	−11	29	90	15	31	85	25	38	78	33	800.2
8	R	24	87	−18	29	85	4	32	85	12	35	69	25	1,526
Mean		21	90	−7	28	87	10	32	82	23	36	74	31	1,273.63

ATA, angle of the two arms; MPTA, medial proximal tibial angle; MSA, medial slope angle; RS, residual stress.

In comparison, there was no significant difference in any of the baseline angles between the groups. There were significant differences regarding the angle of ATA at 6 weeks (*P* = 0.0223) and 12 weeks (*P* = 0.0180), but there was no significant difference at 18 weeks (*P* = 0.6147). We found no significant difference regarding the angle of MPTA at 0 week (*P* = 0.3935,), 6 weeks (*P* = 0.1255), 12 weeks (*P* = 0.8299), and 18 weeks (*P* = 0.5565) intervals. No significant difference existed regarding the angle of MSA at 0 week (*P* = 0.3121), 6 weeks (*P* = 0.0879), 12 weeks (*P* = 0.1935), and 18 weeks (*P* = 0.3635) interval. We found significant differences in residual stress (*P* = 0.001) and width of perichondrial tissue between the center of the plate and physis (*P* = 0.0001). The Spearman’s correlation coefficients for interrater agreement and intraclass correlation coefficient (ICC) were 0.87 and 0.92, respectively.

## Discussion

Angular deformities of the lower limb are common clinical problems encountered in pediatric orthopedic practices. The disorders are a part of the constellation of developmental orthopedic diseases affecting young patients. The deformities can be either valgus or varus and most commonly affect the knee joint [1-3].

The rational behind the use of staples and conventional tension-band plate and screw system is based on Stevens et al.’s theory [3]. Our newly designed system is also based on the theory. The system can correct angular deformities by arresting growth of the other side of the bone. The plate has two arms and a built-in hinge. Based on the previous studies, we designed the rotation of the two arms ranged from 155° to 170° to better fit the contour of the physis in all stages of angular correction. That automatic change can also disperse repeated stress on the surface of periosteum and perichondrium during walking. Therefore, the risk of fixation failure can be reduced. Because of a better match between the implant and bone surface, residual stress at the rim of metaphyseal screw hole was lower after implant removal.

Our histological study showed that the periosteum and perichondrium beneath the hinged plate were intact, but the structures beneath the conventional plate were almost vanished. Aykut et al. [14] verified that healthy periosteal and perichondral were critical for preserving vigor of the growth plate. Our plate with a compression-free hinge may be more appropriate for protecting the growth plate.

Compare to the conventional tension-band plate and screw system, our system has advantages. The self-changeable arc of the two arms produces uniform stress distribution on the growth plate, which may protect the growth plate and perichondrial ring from compressive injuries. The self-changeable arc may also reduce stress concentration on the screws, which decreases the risk of implant failure. The disadvantage of our system is that there is a prominence owing to the bulged hinge, but the morbidity is minimal. Our system can be an alternative to the conventional tension-band plate and screw system, especially in obese patients and patients with poor bone quality such as Blount’s disease. It seems no contraindication compare to the conventional technique.

Our study has limitations. The small sample size affects the reliability of the results because it leads to a high variability. Our assessments on stress distribution and residual stress are based on the changes on images rather than direct measurements, which may not accurately reflect the inherent stress.

## Conclusion

Compared to the conventional tension-band plate and screw system, the use of the hinged plate and screw system may be a more reliable technique with minimal complications for correction of angular deformities of the lower limb.

## Abbreviations

CP: Conventional plate
HP: Hinged plate
MSA: Medial slope angle
MPTA: Medial proximal tibial angle
ATA: Angle of the two arms
